# Adherence to insulin therapy and associated factors among patients with diabetes mellitus in public hospitals of Central Zone of Tigray, Ethiopia, 2018: a cross-sectional study

**DOI:** 10.11604/pamj.2019.33.309.17547

**Published:** 2019-08-20

**Authors:** Teklewoini Mariye, Alem Girmay, Tsyion Birhanu, Hagos Tasew, Girmay Teklay, Zeray Baraki, Hadgu Gerensea, Tewolde Teklu, Gebrewahed Bezabeh

**Affiliations:** 1 Department of Adult Health Nursing, Aksum University, Tigray, Ethiopia; 2 Department of Pediatric Nursing, Aksum University, Tigray, Ethiopia; 3 Department of Pharmacy, Aksum University, Tigray, Ethiopia; 4 Department of Adult Health Nursing, Mekelle University, Tigray, Ethiopia

**Keywords:** Adherence, diabetes mellitus, insulin therapy, Ethiopia

## Abstract

**Introduction:**

Adherence to insulin therapy is a critical factor for adequate control of diabetes mellitus. Despite the multiple well-known benefits of adherence to insulin therapy, poor adherence remains to be a common cause of diabetes mellitus-related complications. A better management of diabetes mellitus requires determining the level of patient adherence and identifying why non-adherence to insulin therapy occurs. Therefore, this study was designed to assess the level of adherence to insulin therapy and associated factors among diabetes mellitus patients.

**Methods:**

The study was conducted from May 1 to July 1, 2018, using a cross-sectional study design. Interviewer-administered questionnaire was employed for data collection and systematic random sampling technique was used to select study participants. The collected data were entered using Epi data version 3.1.1 and exported to SPSS version 22 for analysis. Logistic analysis was carried out to check the level of association between adherence to insulin therapy and the independent variables with significance level of 0.05 at 95% confidence interval.

**Results:**

273 respondents were selected with a 100% response rate. Near to one-fourth (24.2%) of the respondents were adherent to their insulin therapy. The study revealed that good knowledge of diabetes mellitus [AOR=6.51; 95% CI [1.58, 26.71], age [>30 years] [AOR=2.63; 95% CI [1.27, 5.42], knowledge regarding insulin self-injection [AOR=4.21; 95%CI [1.06,16.65], favorable attitude towards insulin injection [AOR=2.14; 95% CI [1.04,4.41], free-of-cost insulin therapy [AOR= 4.62, 95% CI [1.06,16.65], having of glucometer at home [AOR= 2.82, 95% CI [1.12,7.09], and being a member of Ethiopian diabetic association [AOR= 5.41, 95% CI [2.31,12.64] were found to significantly affect adherence to insulin therapy.

**Conclusion:**

Nearly one-fourth of the study participants were adherent to their insulin therapy. Good knowledge and favorable attitude towards insulin injection, good knowledge regarding diabetes mellitus, being a member of the Ethiopian Diabetes Association, age greater than thirty years old, free-of-cost insulin therapy and having glucometer at home were found to be significant predictors of adherence to insulin therapy.

## Introduction

Diabetes mellitus (DM) is a group of metabolic disorder characterized by elevated blood glucose levels (hyperglycemia) [1]. It is one of the major public health problems affecting over 451 million people globally and with a projected increase to 693 million by 2045 [2]. Inadequate control of blood glucose level can lead to several disorders, including cardiovascular diseases, which are the leading causes of morbidity and mortality for people with diabetes mellitus. Moreover, it accounts for the higher direct and indirect medical expense of people affected by DM [3]. To control the burden of DM, patients need to use insulin therapy as ordered by the health care providers. Type 1 DM (T1DM) patients are treated by multiple-dose insulin injection or continuous subcutaneous insulin infusion, but type 2 DM (T2DM) is a progressive disease with a treatment protocol adjusted in line with the disease's progression. Many patients with T2DM eventually require insulin to maintain adequate glycemic control [4]. With the ever-increasing number of DM patients and its negative effect on economy, health system and society, there has been a need for rapid progress in the care and management of patients with insulin treatment which, by improving glycemic control, slows down the progress of microvascular and macrovascular complications [5,6]. Anti-diabetic medications are effective only when they are taken according to the recommendations of health care providers. However, proper orders without good adherence would not result in the intended treatment outcome [7,8]. Good adherence to insulin treatment, compared to a low-level of adherence, has been associated with improved levels of glycemic control [9] which, in turn, reduces the risk of developing acute and chronic diabetic complication [10,11] and mitigating its economic burden [12,13].

Despite the widely known fact of multiple benefits of adherence to insulin therapy, poor adherence among patients remains a common problem in substantial number of patients with diabetes mellitus [14-17]. Specifically, while insulin is the most efficient glucose lowering agent [18], previous researches have indicated that a large proportion of T2DM patients discontinue their basal insulin therapy [16,19,20]. In general, 41-57% of insulin-treated persons were non-adherent with a 20% non-adherence rate [14]. Consequently, a significant number of patients die prematurely from cardiovascular diseases [21]. In addition, some studies in Ethiopia shows that there is poor adherence to insulin therapy among DM patients [22,23]. To improve patient's adherence to insulin treatment, it is important to determine the extent of patient adherence and why non-adherence to insulin therapy occurs. Identifying predictors of poor adherence are necessary to enable stakeholders to take all the initiatives to tackle these factors. However, there is a lack of information about the extent of adherence and associated factors to poor adherence for insulin therapy in developing countries. In Ethiopia, the level of adherence and associated factors of insulin therapy remain to be insufficiently studied. Therefore, this study was designed to assess the level adherence to insulin therapy and associated factors among patients with DM in public hospitals of central zone of Tigray regional state, Ethiopia.

## Methods

The study was conducted in public hospitals of the Central Zone, Tigray Regional State, Ethiopia with a cross-sectional design from May 1, 2018 to July 1, 2018. All DM patients who visit general public hospitals of the central zone of Tigray were the source population. The study population included all diabetic patients who visited general public hospitals during data collection period. Patients who were pregnant, patients under the age of 18 years, newly diagnosed DM patients, patients with severe mental illness, critically ill patients and patients who were not taking insulin therapy were excluded from this study. The sample size was calculated using a single population proportion with EPI Info software version 7.1.1 with the following parameters: confidence level, 95%; marginal error (d), 5%; and the level of adherence to insulin therapy, 66.9%. The level of adherence to insulin therapy was taken from a study conducted at Tikur Anbessa Specialized Hospital in Ethiopia in 2015 [23]. Finally, by adding a non-response rate of 10%, the total sample size was found to be 273. Participants were selected using systematic random sampling technique. The participants were selected from each hospital in accordance with the magnitude of the population they entertain. Lottery method was used to select participants from each hospital.

Data were collected using interviewer-administered structured questionnaire. The questionnaire contained six parts: the first part (part I) was used to collect socio-demographic data, part II was used to collect adherence to insulin therapy, part III was used to collect health profile data of the study subjects, part IV was regarding Diabetes Knowledge Questionnaire (DKQ), part V was knowledge regarding insulin therapy and part VI was used to measure the patients' attitude towards insulin therapy. The data were collected using a structured interviewer-administered questionnaire. A modified morisky adherence scale of 8 items (MMAS-8) was used to measure patients' adherence to their insulin therapy. MMAS-8 is a validated tool which consists of 8 item self report measure of adherence. Items 1 through 7 had response choices of "yes" or "no" whereas item 8 had 5-point Likert scales. Each "no" response was rated as "1" and each "yes" was rated as "0" except for item 5 (reversed score), in which the response "yes" was rated as "1" and "no" was rated as "0". The 5-point Likert scales of item 8, which is concerned on the difficulty to remember taking their insulin therapy, were never/rarely = 0, once in a while = 1, sometimes = 2, usually = 3 and all the time = 4. In this scale, if patients choose response "0", the score is "1" and if they choose response "1,2,3, or 4", the score is "0". Higher scores indicate higher adherence levels. The total scores of all the items range from 0 to 8 and was grouped into two levels: adherent (score of 6 to 8), and non-adherent (score < 6).

Knowledge regarding insulin therapy and knowledge regarding DM had 11 and 24 items, respectively, and each had responses of yes or no [24,25]. The attitude section of the questionnaire had five Likert scales ranging from 1 to 5 and the values were reversed for negative statements. The attitude and knowledge section of the questionnaire were tested for reliability and validity and were also used in Ethiopia in similar study [26,27]. The questionnaire was initially prepared in English and then was translated into the local language (Tigrigna) by Tigrigna and English language experts. To ensure consistency, the Tigrigna questionnaire was again translated back to English by a different language expert. To assess its validity and reliability, the questionnaire was pretested in 10% of the sample size in a different institution, not included in the main study [23,28]. The data were collected by four trained B.Sc. nurses and supervised by two others. Clarity and completeness of the filled questionnaire were checked daily during data collection. Then, it was coded, entered into Epi-Data version 3.1 and exported to SPSS version 22 for analysis. Descriptive statistics were computed to show frequency, percentage distribution, mean and standard deviation. A bivariable logistic regression analysis was conducted to determine the association between the explanatory and outcome variables. Henceforth, multivariable logistic regression analysis was conducted by selecting only variables with a P value ≤ 0.2 in the bivariable analysis. To check for the fitness of the logistic regression model, the Hosmer-Lemeshow test was used. Multicollinearity was assessed by variance inflation factor and all assumptions of binary logistic regression were checked. A statistical analysis was made at a confidence interval of 95% with P value less than 0.05.

### Operational definitions

**Adherence:** is the extent to which a person's behavior; taking medication, following a prescribed diet, and/or executing lifestyle changes corresponds with agreed recommendations from the health care provider. This was measured with Morisky's 8- item scale which, when the MMAS-8 score is ≥ 6, the study participant is considered to be adherent.

**Non-adherence:** is the extent of deviation of drug-taking behavior of a patient from the agreed recommendations by a health care provider. This was measured with Morisky's 8- item scale in which, when the MMAS-8 score is <6, the study participant is considered as non-adherent [29].

## Results

**Demographic profile of study participants:** a total of 273 respondents were selected in this study with a response rate of 100%. Of these study subjects, 37.4% were female participants and more than half of them (59%) were living in rural areas. Close to one-third of the subjects (36.6%) were single and with respect to their educational level, few (16.5%) couldn't read and write. Regarding ethnicity and religion, the majority were Tigrayans (98.9%) and Orthodox Christian (92.7%) (Table 1).

**Table 1:** socio-demographic profile of study population of a study conducted on adherence to insulin and associated factors among patients with diabetes mellitus in public hospitals of Central Zone, Tigray, Ethiopia, 2018

Variables	Non-adhered	Adhered	Total
**Sex**			
Male	138(50.5%)	33(12.1%)	171(62.6%)
Female	69(25.3%)	33(12.1%)	102(37.4%)
**Age**			
18-31	101(37%)	19(7%)	120(44%)
31-55	89(32.6%)	37(13.6%)	126(46.2%)
≥56	17(6.2%)	10(3.7%)	27(9.9%)
**Residence**			
Urban	83(30.4%)	29(10.6%)	112(41%)
Rural	124(45.4%)	37(13.6%)	161(59%)
**Marital status**			
Married	106(38.8%)	46(16.8%)	152(55.7%)
Single	86(31.5%)	14(5.1%)	100(36.6%)
Widowed	3(1.1%)	2(0.7%)	5(1.8%)
Divorced	12(4.4%)	4(1.5%)	16(5.9%)
**Educational level**			
Cannot read and write	31(11.4%)	14(5.1%)	45(16.5%)
Can read and write	12(4.4%)	7(2.6%)	19(7%)
Primary school	85(31.1%)	23(8.4%)	108(39.6%)
Secondary school	61(22.3%)	17(6.2%)	78(28.6%)
Colleague and above	18(6.6%)	5(1.8%)	23(8.4%)
**Occupation**			
House wife	17(6.2%)	7(2.6%)	24(8.8%)
Governmental employee	23(8.4%)	4(1.5%)	27(9.9%)
Private employee	48(17.6%)	16(5.9%)	64(23.4%)
Daily worker	51(18.7%)	12(4.4%)	63(23.1%)
Farmer	68(24.9%)	27(9.9%)	95(34.8%)
**Ethnicity**			
Tigray	205(75.1%)	65(23.8%)	270(98.9%)
Amhara	2(0.7%)	1(0.4%)	3(1.1%)
**Religion**			
Orthodox	193(70.7%)	60(22%)	253(92.7%)
Muslim	14(5.1%)	6(2.2%)	20(7.3%)

**Health profile of the respondents:** in most of the participants (79.5%), the duration since they were diagnosed with DM was less than 10 years. 10% of the participants were getting insulin therapy free of cost. With respect to family history and having glucometer, 16.8% had family history of DM, and 18% had glucometer at home. Nearly two-third (65.8%) of the respondents attended DM-related education, and more than a quarter (29.3%) of the subjects were member of the Ethiopian Diabetic Association (EDA). Almost all (98.5%) of the respondents had visited healthcare provider regularly (Table 2).

**Table 2:** health profile of study population of a study conducted on adherence to insulin and associated factors among patients with diabetes mellitus in public hospitals of Central Zone, Tigray, Ethiopia, 2018

Variable	Non- adhered	Adhered	Total
**Family history of DM**			
Yes	38(13.9%)	8(2.9%)	46(16.8%)
No	169(61.9%)	58(21.2%)	227(83.2%)
**Attendance of diabetes education sessions**			
Yes	22(8.1%)	6(2.2%)	28(10.3%)
No	185(67.8%)	60(22%)	245(89.7%)
**Membership of EDA**			
Yes	43(15.8%)	37(13.6%)	80(29.3%)
No	92(33.7%)	15(5.5%)	107(39.2%)
I did not know	72(26.4%)	14(5.1%)	86(31.5%)
**Having glucometer at home**			
Yes	137(50.2%)	58(21.2%)	195(71.4%)
No	70(25.6%)	8(2.9%)	78(28.6%)
**Duration since diagnosed diabetic (years)**			
<10	163(59.7%)	54(19.8%)	217(79.5%)
10	11(4%)	5(1.8%)	16(5.9%)
>10	33(12.1%)	7(2.6%)	40(14.7%
**Duration since the start of insulin therapy (year)**			
<5	94(34.4%)	33(12.1%)	127(46.5%)
5-10	82(30%)	26(9.5%)	108(39.6%)
>10	31(11.4%)	7(2.6%)	38(13.9%)
**Dosing schedule of insulin**			
Once	2(0.7%)	1(0.4%)	3(1.1%)
BID	205(75.1%)	65(23.8%)	270(98.9%)
**Having regular follow up**			
Yes	204(74.7%)	65(23.8%)	269(98.5%)
No	3(1.1%)	1(0.4%)	4(1.5%)

**Knowledge and attitude of the respondents:** the majority of the respondents had good knowledge regarding insulin self-injection (94.1%) and diabetes mellitus (82.8%). With respect to attitude towards insulin injection, more than a quarter (27.8%) of the respondents had a favorable attitude (Table 3).

**Table 3:** knowledge and attitude towards insulin therapy among patients with diabetes mellitus in public hospitals of Central Zone, Tigray, Ethiopia, 2018

Variable	Non-adhere	Adhered	Total
**Knowledge regarding Diabetes mellitus**			
Good knowledge	163(59.7%)	63(23.1%)	226(82.8%)
Poor knowledge	44(16.1%)	3(1.1%)	47(17.2%)
**Knowledge regarding Insulin self-injection**			
Good knowledge	199(72.9%)	58(21.2%)	257(94.1%)
Poor knowledge	8(2.9%)	8(2.9%)	16(5.9%)
**Attitude towards insulin injection**	Not adhere	Adhere	Total
Favorable	52(19%)	24(8.8%)	76(27.8%)
Unfavorable attitude	155(56.8%)	42(15.4%)	197(72.2%)

**Factors associated with adherence to insulin therapy:** analysis of the data with multivariable logistic regression showed seven variables to be significantly associated with adherence to insulin therapy at 5% level of significance. Knowledge of insulin self-injection was significantly associated with adherence to insulin therapy. The odds of adherence to insulin therapy among those who had good knowledge of insulin self-injection was 4.21 times greater than those who have lesser knowledge of insulin self-injection [AOR=4.21; 95% CI [1.06, 16.65]. Having glucometer at home showed a significant association with adherence to insulin therapy. The analysis test indicated that respondents who had glucometer at their home were 3 times more likely to adhere to insulin therapy compared to their counter parts [AOR=2.81; 95% CI [1.12, 7.09]. Attitude towards insulin therapy was found to affect adherence. The odds of adherence to insulin therapy were 2.14 times higher among respondents who have favorable attitude towards insulin therapy compared to their counter parts. [AOR=2.14; 95%CI [1.04, 4.41]. Knowledge of DM was found to be the factor having the strongest association. Subjects with good knowledge about DM were 6.51 times more likely to adhere to insulin therapy than those with poor knowledge of DM [AOR=6.51; 95%CI [1.58, 26.72]. Free delivery of insulin was also found to affect adherence in which respondents who got their insulin therapy for free were more likely to adhere to insulin therapy than those who did not [AOR=4.62, 95% CI [1.12, 19.05]. Membership of Ethiopian Diabetes Association (EDA) was significantly associated with adherence to insulin therapy compared to the non-members. The membership resulted in a likelihood of 5.41 times more adherence than those who were not members [AOR=5.41; 95% CI [2.31, 12.64]. The respondents whose age was greater than or equal to 56 and age range of 31-55 years showed better adherence to insulin therapy than those respondents aged less than or equal to 30 years. The odds of adherence to insulin therapy in the subjects aged 56 or more years and those aged 31-55 years were 4.25 and 2.63 times more, respectively, than those ≤ 30 years [AOR=5.41; 95% CI [2.31, 12.78] and AOR=2.63; 95% CI [1.27, 5.41] (Table 4).

**Table 4:** logistic regression analysis of adherence to insulin therapy and associated factors among patients with diabetes mellitus in public hospitals of Central Zone Tigray, Ethiopia, 2018

Variable	Non- adhered	Adhered	COR [95%CI]	p-value	AOR [95%CI]	P-value
**Sex**						
Male	138(50.5%)	33(12.1%)	0.5[0.28,0.87]	**0.16**	0.57[0.30,1.11]	0.10
Female	69(25.3%)	33(12.1%)	1		1	
**Age**						
<31	101(37%)	19(7%)	1		1	
31-55	89(32.6%)	37(13.6%)	2.21[1.18,4.12]		2.63[1.27,5.42]	**0.009**
≥56	17(6.2%)	10(3.7%)	3.13[1.24,7.86]		4.25[1.42,12.78]	**0.01**
**Occupation**						
House wife	17(6.2%)	7(2.6%)	1.04[0.38,2.78]	**0.94**	1.14[0.37,3.48]	0.82
Governmental employee	23(8.4%)	4(1.5%)	0.44[0.14,1.38]	**0.16**	0.99[0.36,2.75]	0.99
Private employee	48(17.6%)	16(5.9%)	0.63[0.84,0.41]	**0.63**	0.30[0.06,1.40]	0.13
Daily worker	51(18.7%)	12(4.4%)	0.59[0.27,1.28]	**0.18**	0.67[0.17,2.57]	0.56
Farmer	68(24.9%)	27(9.9%)	1		1	
**Educational status**						
Cannot read and write	31(11.4%)	14(5.1%)	1		1	
Can read and write	12(4.4%)	7(2.6%)	1.29[0.42,3.98]	**0.65**	1.10[0.24,5.14]	0.90
Primary school	85(31.1%)	23(8.4%)	0.59[0.27,1.31]	**0.19**	0.75[0.25,2.23]	0.60
Secondary school	61(22.3%)	17(6.2%)	0.62[0.26,1.41]	**0.25**	0.78[0.29,2.10]	0.63
Colleague$ above	18(6.6%)	5(1.8%)	0.61[0.19,1.99]	**0.41**	2.21[0.53,9.23]	0.28
**Family History of DM**						
Yes	38(13.9%)	8(2.9%)	1		1	
No	169(61.9%)	58(21.2%)	1.63[0.72,3.69]	**0.19**		
**Membership of EDA**						
Yes	43(15.8%)	37(13.6%)	4.42[2.15,9.11]	**0.00**	5.41[2.31,12.64]	**0.00**
No	92(33.7%)	15(5.5%)	0.84[0.38,1.85]	**0.38**	1.19[0.51,2.81]	
I did not	72(26.4%)	14(5.1%)	1		1	
**Having Glucometer at home**						
Yes	137(50.2%)	58(21.2%)	3.70[1.67,8.18]	**0.01**	2.81[1.12,7.09]	**0.028**
No	70(25.6%)	8(2.9%)	1		1	
**Cost of insulin**						
Free	25(9.2%)	3(4.5%)	2.88[0.84,9.88]	**0.09**	4.62[1.12,19.05]	**0.034**
Purchased	182(66.7%)	63(95.5%)	1		1	**1**
**Attitude towards insulin injection**						
Favorable	52(19%)	24(8.8%)	1.7[0.94,3.07]	**0.07**	2.14[1.04,4.41]	**0.038**
Unfavorable	155(56.8%)	42(15.4%)	1		1	
**Knowledge regarding Insulin self-injection**						
Good knowledge	199(72.9%)	58(21.2%)	3.43[1.23,9.54]	**0.01**	4.21[1.0616.65]	**0.041**
Poor knowledge	8(2.9%)	8(2.9%)	1		1	
**Knowledge regarding DM**						
Good knowledge	163(59.7%)	63(23.1%)	5.67[1.69,18.92]	**0.005**	6.51[1.58,26.72]	**0.009**
Poor knowledge	44(16.1%)	3(1.1%)	1		1	

## Discussion

This study provided an insight into the level of adherence to insulin therapy and associated factors among patients with DM in public hospitals of central zone, Tigray regional state, Ethiopia in the year 2018. The level of adherence to insulin therapy was found to be 24.2%. This was considerably lower than findings from other studies conducted in Tukur Anbesa Specialized Hospital (TASH) and Felege Hiwot Referral Hospital which were 67% [23] and 59.2% [22], respectively. This discrepancy may have resulted because of the difference in lifestyle, sample size, time gap and health service utilization. The knowledge of insulin self-injection was found to significantly associate with adherence to insulin therapy. This association could be due to the fact that having good knowledge helps follow the correct way and time of insulin self-administration in the way prescribed by health care providers [30]. Patients who were having glucometer at home had better adherence to insulin therapy than those who don't. This difference may be related with the convenience of using glucometer at home in which patients frequently check and control their blood glucose level. The result of this regular checkup may have encouraged them to take insulin regularly [31]. Patients with a favorable attitude towards insulin therapy showed better adherence than those with an unfavorable attitude. This result was congruent with the finding of a study conducted at Marmara University [32]. This might be due to a better understanding of the advantage of insulin therapy.

Knowledge regarding DM showed an association with adherence to insulin therapy such that having good knowledge was shown to significantly augment adherence. This was consistent with a report from Baqai Institute of Diabetology & Endocrinology and Diabetes Association of Pakistan [30]. Good knowledge of DM helps patients follow the recommended behaviors such as having glucometer at home, having favorable attitude to insulin therapy and identifying and being cautious about the complication of insulin therapy. Access to free-of-cost insulin was seen to positively associate with adherence to insulin. This was consistent with other studies conducted in TASH and Adama Referral Hospital, Ethiopia [22,33]. This may be related with the low socioeconomic status of study participants which challenges them to afford medication costs on top of their daily living expenses. Being a member of EDA was found to result in 5 times higher adherence compared to those who were not members. This might be because, as the association often provides continuous DM-related education, patients may have a better knowledge of insulin therapy, knowledge of diabetes mellitus and have favorable attitude towards insulin. These factors might have enhanced the likelihood of adherence to treatment [34]. The respondents whose age was greater than or equal to 56 years and with the age range of 31-55 years showed association with adherence to insulin therapy than those aged less than or equal to 30 years. The result is consistent with the finding of a study conducted at Tikur Ambessa Specialized Hospital, Addis Ababa, Ethiopia [35]. This might be related to the better experience that these patients may have acquired over the course of their relatively long treatment.

## Conclusion

Nearly one-fourth of the study participants were found to be adherent to their insulin therapy. Good knowledge and favorable attitude towards insulin therapy, good knowledge regarding diabetes mellitus, being member of a diabetes association, older age (>30 years), getting insulin therapy for free and having glucometer at home were found to be the predictors of adherence to insulin therapy.

### What is known about this topic

- Poor adherence to insulin therapy is the principal cause for the development of diabetes mellitus complications;
- There is a lack of information about the extent of adherence and associated factors to insulin therapy in the developing countries;
- While insulin is the most efficient glucose-lowering agent, previous research has indicated that a large proportion of diabetes mellitus patients discontinue their insulin therapy due to various factors.

### What this study adds

- Nearly one-fourth of the study participants were adherent to their insulin therapy;
- Free-of-cost insulin therapy, having glucometer at home, a good knowledge of DM, favorable attitude towards insulin therapy, being members of diabetes association and older age were the significant predictors of adherence to insulin therapy;
- Therefore, health care professionals must encourage patient to be members of DM association and take actions to increase patient’s knowledge, attitude and practice towards insulin therapy by strengthening the delivery of information, education and communication program.

## Competing interests

The authors declare no competing interests.
